# Extruded disc herniations are experienced earlier by inactive young people in the high-tech gaming era

**DOI:** 10.25122/jml-2021-1059

**Published:** 2021

**Authors:** Demet Ucar, Sedat Duman, Yusuf Bayram, Bekir Yavuz Ucar

**Affiliations:** 1. Department of Physical Treatment and Rehabilitation, Beykent University, School of Health Sciences, Istanbul, Turkey; 2. Department of Orthopaedics and Traumatology, University of Health Sciences, Sancaktepe Training Research Hospital, Istanbul, Turkey; 3. Department of Orthopaedics and Traumatology, Tokat Turhal Government Hospital, Tokat, Turkey; 4. Department of Orthopaedics and Spine Surgery, University of Health Sciences, Umraniye Training Research Hospital, Istanbul, Turkey

**Keywords:** disc herniation, young people, inactive youth

## Abstract

In this study, we would like to draw attention to the advanced disc diseases seen in young people. The objective is to investigate the reasons for the increasing trend of disc herniations in young people. A total of 33 young patients with extruded lumbar disc herniations managed by conservative or surgical approaches between 2017 and 2018 were included. The average patient age was 25 years. Smoking, familial predisposition, sporting activity, and the occupation of the patients were questioned and noted. A visual analog scale (VAS) was used to assess the efficacy of pain. Body mass index (BMI) was calculated. All patients were subjected to lumbar magnetic resonance imaging. Eighteen patients (8 females, 10 males) had disc extrusion at the L5-S1 level, whereas 12 patients (8 females, 4 males) had disc extrusion at the L4-L5 level. Three other patients had disc extrusion at the level of both L4-L5 and L5-S1 levels. Motor deficits were detected in four patients, and surgical treatment was required and performed. The other 29 patients were treated conservatively. Young non-sport-oriented patients may face severe disc herniations. Understanding how spine degeneration can affect the likelihood of developing a herniated disc can help people make small lifestyle changes to postpone any serious pain and deficits. While aging is unavoidable, simple lifestyle changes can help improve overall spine health and deter the risk of developing a degenerative spine condition.

## INTRODUCTION

Spinal disc herniation is a disease in which the outer portion of the vertebral disc is torn, enabling the inner portion to extrude through the spinal canal. It compresses the nerves around the disc in the spinal canal and creates pain. This condition is especially seen with natural degeneration changes. With age, the vertebral discs begin to lose water content and become brittle. They can become herniated with the slightest inconvenience. Unfortunately, disc herniation has begun to appear at a young age in recent years [1–3]. The most probable cause of this situation is the inactivity of young people, leading to the early degeneration of immobile vertebral discs [2]. In this study, we searched for young patients who have developed extruded lumbar disc herniations in the past year and would like to draw attention to the advanced disc diseases seen in young people. Our aim was to investigate the reasons for the ascending trend of disc herniations in young people.

## MATERIAL AND METHODS

This is a prospective analysis of 33 young patients (16 females and 17 males) with extruded lumbar disc herniations managed by conservative (medical and physical therapy) or surgical approaches between 2017 and 2018. The mean age of the participants was 25 years (ranging between 17 and 30). All procedures performed in this study involving human participants were in accordance with the ethical standards of the institutional and national research committee and the 1964 Declaration of Helsinki and its later amendments or comparable ethical standards. Neurological examinations were conducted for each participant. Smoking, familial predisposition, sporting activity, and the occupation of the patients were questioned and noted. A visual analog scale (VAS) was used to assess the efficacy of pain management regimens in patients. Body mass index (BMI) was calculated by dividing the patient’s weight by their height squared and reported as kg/m^2^. BMI was classified as follows: 25 to 30 kg/m^2^ (overweight); 30 to 40 kg/m^2^ (obese); and >40kg/m^2^ (morbidly obese), according to the World Health Organization (WHO) definitions. All patients were also subjected to lumbar magnetic resonance imaging.

## RESULTS

Eighteen patients (8 females, 10 males) had disc extrusion at the L5-S1 level, whereas 12 patients (8 females, 4 males) had disc extrusion at the L4-L5 level. Three other patients had disc extrusion at the level of both L4-L5 and L5-S1 levels. Motor deficits were detected in four patients, and surgical treatment was required and performed. The other 29 patients were treated conservatively. All data are shown in Table 1.

**Table 1:** Data of all the participants included in the study.

Patient no.	Age	Sex	Occupation	Smoking	Familial predisposition	Level	VAS	Treatment	VAS (follow-up)
**1**	30	F	Office worker	Yes	Yes	L4-L5	8	Conservative	4
**2**	29	M	Banker	No	Yes	L5-S1	7	Conservative	4
**3**	24	F	Student	No	Yes	L5-S1	8	Conservative	3
**4**	30	M	Driver	Yes	No	L5-S1	6	Conservative	3
**5**	27	M	Secretary	Yes	Yes	L5-S1	9	Conservative	3
**6**	26	M	Driver	Yes	Yes	L4-L5	6	Conservative	2
**7**	26	M	Banker	No	No	L5-S1	7	Conservative	2
**8**	23	F	Student	No	Yes	L5-S1	8	Conservative	3
**9**	22	M	Student	No	Yes	L4-L5 + L5-S1	8	Conservative	2
**10**	17	F	Student	No	Yes	L5-S1	6	Conservative	2
**11**	20	F	Student	No	No	L5-S1	8	Conservative	4
**12**	26	M	Office worker	Yes	No	L5-S1	7	Conservative	2
**13**	24	F	Student	Yes	Yes	L4-L5	7	Conservative	3
**14**	28	F	Nurse	Yes	Yes	L4-L5	7	Conservative	4
**15**	29	M	Banker	Yes	No	L5-S1	8	Conservative	2
**16**	30	M	Office worker	Yes	Yes	L5-S1	8	Conservative	3
**17**	25	M	Office worker	Yes	Yes	L5-S1	6	Conservative	3
**18**	24	M	Student	Yes	Yes	L4-L5 + L5-S1	7	Conservative	3
**19**	27	F	Banker	Yes	No	L4-L5	8	Surgery	2
**20**	26	F	Unemployed	Yes	Yes	L5-S1	6	Conservative	2
**21**	24	M	Driver	Yes	Yes	L5-S1	6	Conservative	2
**22**	26	M	Secretary	Yes	No	L4-L5	8	Conservative	3
**23**	19	F	Student	No	Yes	L4-L5	5	Conservative	3
**24**	25	F	Nurse	No	Yes	L5-S1	7	Conservative	2
**25**	23	M	Student	No	No	L4-L5	6	Conservative	2
**26**	18	F	Student	No	Yes	L4-L5	6	Conservative	2
**27**	19	F	Student	No	No	L4-L5	9	Surgery	2
**28**	17	M	Student	No	Yes	L4-L5 + L5-S1	8	Surgery	2
**29**	29	M	Office worker	Yes	Yes	L5-S1	5	Conservative	3
**30**	28	M	Office worker	Yes	No	L4-L5	6	Conservative	2
**31**	29	F	Housewife	Yes	Yes	L5-S1	7	Conservative	4
**32**	30	F	Secretary	Yes	Yes	L4-L5	7	Surgery	2
**33**	30	F	Housewife	Yes	Yes	L5-S1	6	Conservative	2

F – female; M – male; VAS – visual analog scale.

When the occupations of the patients were examined, it was observed that they all sat during the day and were deprived of movement. The number of smokers was 20 (61%). The mean body mass index value was 32.5 kg/m^2^. The minimum follow-up was 6 months, with an average of 12 (ranging from 6 to 18 months).

**Figure 1:**
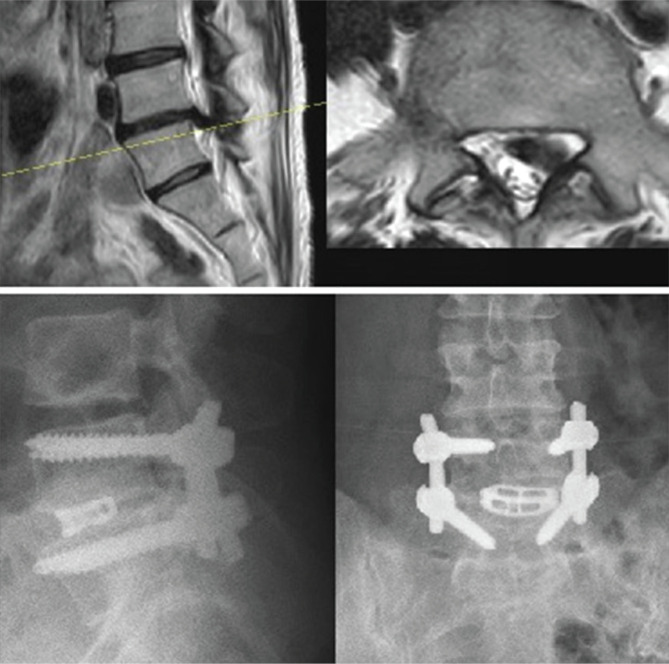
Preoperative magnetic resonance images and postoperative X-ray images.

**Figure 2:**
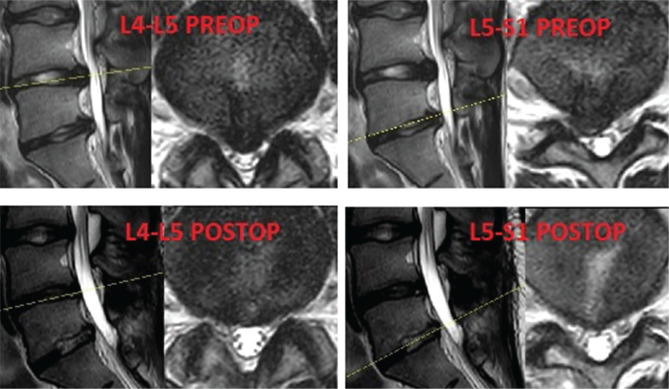
Preoperative and postoperative magnetic resonance images.

Of the patients who underwent surgery, three procedures involved the L4-L5 level. One of them had right L5 radiculopathy and motor deficit, while the others were on the left side. Also, they had L5 sensory impairment and coronal imbalances. Discectomy and interbody fusion surgeries were performed on these patients (Figure 1). Another patient who had severe pain and two motor deficits had surgery at two levels: L4-L5 and L5-S1. Only discectomies were performed on this patient (Figure 2). No complications occurred in the study group.

Physical therapy was performed in the case of patients treated conservatively; they were also given pain medication. For these patients, who have not developed sporting activities, spine ergonomics were explained, and regular walks were started. They were encouraged to lose weight with the help of dietitians, and smoking cessation therapy was started for smokers. None of the patients had motor deficits, and the pain was diminished. The VAS scores were statistically reduced during the follow-up (p<0.05), and all patients have lost weight. During the final follow-up, it was understood that all the patients had started their regular sporting activities, walked for one hour daily, and paid attention to ergonomics. Unfortunately, only three patients quit smoking.

## DISCUSSION

As we age, the intervertebral discs begin to dehydrate and lose their height and shape, and the elasticity in the outer layer of the discs begins to weaken, again causing the discs to lose shape. However, these damaged discs that develop while aging now appear more often in young patients [1–4].

Various age-independent risk factors can lead to unnecessary pressure on discs, which can alter the elasticity in the discs and cause them to lose shape. These risks include smoking, inactivity, obesity, familial predisposition, weak core muscles, and improper lifting [5–8]. Unfortunately, many members of the young generation have developed smoking habits and are inactive, obese, and weak. Therefore, disc disease, which has many risk factors, is inevitable in these young people [1, 2]. In our study, none of our patients was actively involved in sports. They tended to spend most of the day sitting down. A research study about disc herniations in astronauts showed that muscle weakness and/or dysfunction may increase the injury risk and may also predispose to herniation. It has also been established that muscle atrophy occurs around the lumbar spine during space flights [8]. It seems that young non-sport-oriented patients may face similar issues.

The patients in our study also had a higher BMI. The mean value was 32.5 kg/m^2^, which is considered overweight. Madsbu *et al*. showed a higher risk of lumbar disc herniation in obese patients [5]. Pietila *et al*. reported that the patients in their study suffering from lumbar herniation had higher BMIs compared to a randomly selected group of individuals of similar age [7]. This finding suggests that the patient’s physical constitution is an important factor, as increasing body weight results in increased load to the spinal column.

Occupational low back pain and disc diseases are a great burden for the healthcare system [9–12]. In industrialized countries, low back pain and sciatica represent two of the leading causes of work incapacity and disability before the age of 45 [13]. An analysis of several longitudinal studies has confirmed the existence of a strong relationship between certain job postures and the incidence of low back pain (manual handling of loads, leaning forward or backward, twisting of the trunk, and whole-body vibrations); it was also noted that some psychosocial factors, such as social support at work, can also play an important role [9, 14]. In our study, certain occupations seemed to be the cause of postural disturbances in the patients: secretary work environment, driver, office worker, student, banker, nurse, and housewife.

Recent studies have proved that a family history of lumbar disc herniation has a significant implication [7, 15, 16]. In one study, valid family history was obtained for 121 patients, and a positive history was found in 82 of these patients (67.8%) [7]. Another study reported that the relative risk of development of herniation of a lumbar disc before the age of 21 years was estimated to be approximately five times greater in patients with a positive family history [16]. Our study generated similar results; we found familial predisposition in 23 patients (69%). Genetic factors may also be involved in the development of lumbar disc herniation as an expression of degeneration. However, further studies are needed in this regard.

Many patients who develop a herniated disc can manage symptoms using conservative treatment, such as pain medication and physical therapy [17, 18]. These treatments help relieve pressure on the nerve root and increase circulation to the disc, so the body can begin to heal itself through the process of resorption. However, if chronic pain persists after conservative treatment and/or if a motor deficit is identified, surgery should be recommended. In our study, only four patients had surgery because of motor deficits. Fully neurological recovery postoperatively was seen in all of them.

Spinal interbody fusion is a potential option in patients with primary disc herniations who have significant chronic axial back pain and bi-radicular symptoms and practice manual labor daily [19]. Advocates for fusion during the discectomy argue that segment stabilization may prevent late-onset instability and the development of chronic low back pain. In our study, discectomy and interbody fusion surgeries were performed on two patients. They had L5 sensory impairment, coronal imbalances and chronic axial low back pain.

## CONCLUSION

Understanding how spine degeneration can affect the likelihood of developing a herniated disc could help people make small lifestyle changes to postpone any serious pain and deficits. While aging is unavoidable, simple lifestyle changes can help improve overall spine health and deter the risk of developing a degenerative spine condition.

## References

[1] Fei H, Li WS, Sun ZR, Ma QW, Chen ZQ (2017). Analysis of Spino-pelvic Sagittal Alignment in Young Chinese Patients with Lumbar Disc Herniation. Orthop Surg.

[2] Karademir M, Eser O, Karavelioglu E (2017). Adolescent lumbar disc herniation: Impact, diagnosis, and treatment. J Back Musculoskelet Rehabil.

[3] Dewing CB, Provencher MT, Riffenburgh RH, Kerr S, Manos RE (2008). The outcomes of lumbar microdiscectomy in a young, active population: correlation by herniation type and level. Spine.

[4] Ozgen S, Konya D, Toktas OZ, Dagcinar A, Ozek MM (2007). Lumbar disc herniation in adolescence. Pediatr Neurosurg.

[5] Madsbu MA, Øie LR, Salvesen Ø, Vangen-Lønne V, Nygaard ØP, Solberg TK, Gulati S (2018). Lumbar Microdiscectomy in Obese Patients: A Multicenter Observational Study. World Neurosurg.

[6] Madsbu MA, Salvesen Ø, Werner DAT, Franssen E, Weber C, Nygaard ØP, Solberg TK, Gulati S Fukuda S, Susa M, Watanabe I, Nishimoto K, Horiuchi K, Toyama Y, Morioka H (2018). Surgery for Herniated Lumbar Disc in Daily Tobacco Smokers: A Multicenter Observational Study. World Neurosurg.

[7] Pietilä TA, Stendel R, Kombos T, Ramsbacher J, Schulte T, Brock M (2001). Lumbar disc herniation in patients up to 25 years of age. Neurol Med Chir.

[8] Belavy DL, Adams M, Brisby H, Cagnie B, Danneels L, Fairbank J, Hargens AR, Judex S, Scheuring RA, Sovelius R, Urban J, van Dieën JH, Wilke HJ (2016). Disc herniations in astronauts: What causes them, and what does it tell us about herniation on earth?. Eur Spine J.

[9] Petit A, Roquelaure Y (2015). Low back pain, intervertebral disc and occupational diseases. Int J Occup Saf Ergon.

[10] Ahsan MK, Matin T, Ali MI, Ali MY, Awwal MA, Sakeb N (2013). Relationship between physical work load and lumbar disc herniation. Mymensingh Med J.

[11] Kara B, Tulum Z, Acar U (2005). Functional results and the risk factors of reoperations after lumbar disc surgery. Eur Spine J.

[12] Uçar D, Bozkurt M, Uçar BY, Bulut M, Azboy I (2011). Chronic low back pain in housewives. J Clin Exp Invest.

[13] European Agency for Safety and Health at Work (2010). OSH in figures: work-related musculoskeletal disorders in the EUFacts and figures. Luxembourg.

[14] Hoogendoorn WE, van Poppel MN, Bongers PM, Koes BW, Bouter LM (1999). Physical load during work and leisure time as risk factors for back pain. Scand J Work Environ Health.

[15] Matsui H, Kanamori M, Ishihara H, Yudoh K, Naruse Y, Tsuji H (1998). Familial predisposition for lumbar degenerative disc disease. A case-control study. Spine.

[16] Varlotta GP, Brown MD, Kelsey JL, Golden AL (1991). Familial predisposition for herniation of a lumbar disc in patients who are less than twenty-one years old. J Bone Joint Surg Am.

[17] Demirel A, Yorubulut M, Ergun N (2017). Regression of lumbar disc herniation by physiotherapy. Does non-surgical spinal decompression therapy make a difference? Double-blind randomized controlled trial. J Back Musculoskelet Rehabil.

[18] Zhang B, Xu H, Wang J, Liu B, Sun G (2017). A narrative review of non-operative treatment, especially traditional Chinese medicine therapy, for lumbar intervertebral disc herniation. Biosci Trends.

[19] Çaçan MA, Uçar BY (2019). What every spine surgeon should know about transforaminal lumbar interbody fusion surgery for herniated discs. International Orthopaedics.

